# Evolution of Rabies in South America and Inter-Species Dynamics (2009–2018)

**DOI:** 10.3390/tropicalmed6020098

**Published:** 2021-06-09

**Authors:** Mauro Meske, Angela Fanelli, Felipe Rocha, Lina Awada, Paula Caceres Soto, Neo Mapitse, Paolo Tizzani

**Affiliations:** 1 OIE—World Organisation for Animal Health, 75017 Paris, France; m.meske@oie.int (M.M.); l.awada@oie.int (L.A.); p.caceres@oie.int (P.C.S.); n.mapitse@oie.int (N.M.); 2 Department of Veterinary Medicine, University of Bari, 70121 Bari, Italy; angela.fanelli@uniba.it; 3 PAHO-WHO-PANAFTOSA-Centro Panamericano de Fiebre Aftosa y Salud Pública Veterinaria, Regional Information System for the Epidemiological Surveillance of Rabies (SIRVERA), 25045-002 Duque de Caixas, Brazil; rochafe@paho.org

**Keywords:** rabies, veterinary public health, surveillance, livestock, vampire bat, *Desmodus rotundus*, infectious diseases, wildlife disease, dog-mediated rabies, OIE-WAHIS, SIRVERA

## Abstract

Rabies is listed as one of the World Health Organisation’s (WHO) Neglected Tropical Diseases Worldwide, with a significant impact in South America. This paper explores the dynamics of rabies cases in humans, pets (dogs and cats), livestock and wildlife (bats in particular) in South America during the period 2009–2018. The data used in this study were derived from the two main databases for rabies in South America: the OIE-WAHIS from the World Organisation for Animal Health (OIE) and PANAFTOSA’s Regional Information System for the Epidemiological Surveillance of Rabies (SIRVERA). Being a neglected disease with possible underreporting in some areas, the reported rabies cases may not always represent the real disease burden. The analysis focuses on the evolution of the number of cases in time and their spatial distribution, as well as on the main source of infections in humans, determined by laboratory assays of the antigenic variant or through epidemiological investigations. Additionally, Generalised Linear Mixed Models (GLMM) were used to evaluate the risk factors associated with the occurrence of human cases. Our results show that the highest impact of the disease in terms of number of cases was reported on livestock, while the overall number of cases (in animals and humans) progressively decreased along the study period. The spatial distribution of rabies in livestock showed two main clusters in the north-western (mainly Colombia) and in the south-eastern part of the affected area (Brazil), and a third smaller cluster in Peru. A cluster in dogs was observed in Bolivia. Out of the 192 human cases reported during the study period, 70% of them were transmitted by bats. The number of human cases reported during the study period were significantly associated with the number of rabies cases reported in livestock, pets and wildlife. Despite the overall decreasing case report rate, the disease still represents a major animal and public health concern in South America, and new strategies for compiling systematic information, networking and education are needed, as well as the education and training of veterinary staff.

## 1. Introduction 

Rabies is a viral infection caused by negative-sense single stranded RNA viruses belonging to the genus *Lyssavirus* (family *Rhabdoviridae*). Within the genus, the International Committee on Taxonomy of Viruses (ICTV) [1] delineated different virus species, all causing fatal encephalitis in humans and other mammals. The prototype member of the genus is the rabies virus species (RABV), which is the most important from an epidemiological perspective for animal and public health [2]. RABV is typically transmitted through the bite of a rabid animal. Other transmission methods include the inhalation of viruses’ particles, transplants of organs, and infection of mucous membranes (in case of open wounds/abrasions) with saliva and brain tissue from a rabid animal containing RABV [3]. Several mammals, mainly belonging to the orders *Carnivora* and *Chiroptera*, serve as major hosts in different parts of the world. Overall, two major epidemiological cycles of rabies exist: the urban cycle, in which dog is the principal reservoir, and the epidemiological sylvatic cycles (terrestrial—maintained by terrestrial animals, like foxes, raccoons, wild *carnivores*—and aerial—maintained by bats in general) [4].

Rabies is listed as one of the World Health Organisation’s (WHO) Neglected Tropical Diseases and Worldwide, with a significant disease burden, especially in Africa and Asia [5]. Oral Rabies vaccine programs have been employed in North America to deliver oral rabies vaccines bait to wildlife. These programs have successfully eliminated fox rabies in several countries in Europe, reduced the virus significantly in Ontario and eliminated coyote rabies in southern Texas [6]. In Latin America, the common vampire bat (*Desmodus rotundus*), a hematophagous bat*,* is the major sylvatic rabies reservoir and a source of infection for humans and livestock [7]. The common vampire bat is spread throughout South America (except in the south of Patagonia region) to northern Mexico [7,8]. Rabies still remains a serious problem in South America where the two main epidemiological cycles overlap [1], even if the overall epidemiological situation in the Region has significantly improved. To reduce the burden of dog-mediated rabies, the Pan American Health Organization (PAHO) introduced in 1983 a regional control programme which led to a dramatic decrease of 95% of rabies cases in humans and 98% in dogs, thanks to a large-scale dog vaccination and improved access to pre-and post-exposure prophylaxis, as well as higher quality vaccines, and improvement of surveillance and diagnosis. Nevertheless, four human cases of dog-mediated rabies were recently reported in 2021 in Cochabamba, Bolivia [9]. Based on the results achieved in the past 36 years, PAHO expects to eliminate human rabies of canine origin (antigenic rabies variants 1 and 2) from the American Continent by 2022 [10,11]. However, programmes for control of rabies in wildlife, are still not well established, mainly due to financial constraints. For these reasons, several cases of transmission from the common vampire bats to humans and livestock are still registered in South America [11,12]. Human activities have contributed to boost the common vampire bat densities and distribution, through abundant food supply (cattle ranching) as well as with roosting sites in the form of buildings, bridges, and wells. This has increased the chances of rabies transmission. It is very likely to find a higher incidence of rabies in areas where livestock is well established, where ecological characteristics (proximity of a river, forest, crop cultivation) and abundance of food allow the establishment of large colonies of the common vampire bats and higher connectivity between populations [13,14,15]. The deforestation of large areas, which represents a growing problem in many South American countries, provokes the dispersion of the common vampire bats in search of a new location to colonize and may favour the contact between humans and bats [16,17]. The subsequent establishment of large cattle farms in those areas could increase the future incidence of cases of rabies in livestock and humans [18,19].

Rabies is an OIE-listed disease, with the obligation for OIE Member to report the information on the occurrence of the disease in animals [20]. According to the OIE *Terrestrial Animal Health Code*, a rabies case is “any animal infected with rabies virus” and “dog-mediated rabies is defined as any case caused by rabies virus maintained in the dog population (*Canis lupus familiaris*) independently of other animal reservoir species, as determined by epidemiological studies”. The information submitted to the OIE by Member Countries is publicly available online on the World Animal Health Information System (OIE-WAHIS): https://wahis.oie.int/#/home (accessed on 3 June 2021). The World Organisation for Animal Health (OIE) also leads international efforts to combat the disease, providing science-based standards regarding (a) the diagnosis of rabies, (b) the production of high-quality veterinary vaccines, (c) guidelines and recommendations for the control of the disease in animals. Additionally, OIE, WHO, Food and Agriculture Organization of the United Nations (FAO) and Global Alliance for Rabies Control (GARC) are working together in the framework of a comprehensive Strategic Plan to reach zero human dog-mediated rabies cases by 2030 (“Zero by 30”). The plan aims to provide a coordinated common ground for rabies prevention, through the strengthening of human and veterinary health systems. Considering that, despite the enormous progress in the last decades, the burden of rabies is still significant in South America, this work used the official information retrieved from the OIE-WAHIS database to describe: (i) the dynamics of rabies cases in animals and humans from 2009 to 2018; (ii) the spatial case report rate at administrative division level, (iii) the source of human cases and (iv) the factors affecting the occurrence of human cases in South America.

Previous studies have performed descriptive and statistical analysis to summarize data reported by the National Veterinary Services to the OIE, providing valuable insights on the epidemiological situation of the diseases at both regional and global scale [21,22,23]. In this study, to complement OIE-WAHIS data, human and animal rabies cases, including epidemiological details (e.g., whether it was linked to the aerial or terrestrial cycles variants), were extracted from the Regional Epidemiologic Surveillance System for Rabies (SIRVERA), maintained by the Veterinary Public Health area of PAHO and available online: https://sirvera.panaftosa.org.br/ (accessed on 3 June 2021).

## 2. Material and Methods

### 2.1. Study Area

The analysis included data on animal and human cases, submitted by the 13 countries included in the South America Region to both OIE-WAHIS and SIRVERA systems, during the period 2009–2018. The study area included 239 first level administrative divisions, representing the most accurate level of spatial data provided. The area of study (countries of South America) was chosen due to the geographical distribution of *Desmodus rotundus* and considering the completeness of the available data.

### 2.2. Databases Used and Species Categorization

Data were collected from the World Organisation for Animal Health (OIE) and the Centro Panamericano de Fiebre Aftosa, Salud Publica Veterinaria (PANAFTOSA). OIE’s mandate is to ensure transparency in the global animal disease situation. These data are submitted to the OIE by the National Authorities of 182 OIE Member Countries that have the legal obligation to report data concerning notifiable animal diseases, including rabies. An additional 20 countries and territories provide information to the OIE on a voluntary basis. All countries of South America provide information to the OIE. The data used in this study were derived from the OIE monitoring system, that includes data sent every six months by each country on the absence or presence, changes in the occurrence of all OIE-listed diseases in animals, and related quantitative data. PANAFTOSA’s Regional Information System for the Epidemiological Surveillance of Rabies (SIRVERA) [9] collects monthly data on the occurrence of rabies in animals and humans in countries of Latin America. It also collects information on the source of infection for human cases.

The information collected from the two databases were merged and integrated, to increase the accuracy and exhaustiveness of the information available on humans and animals related to rabies. In our study, the information on the number of rabies cases in animals per year and by species for the first administrative divisions of the 13 countries under study was collected mainly from OIE-WAHIS. On the other hand, the number of rabies cases in humans and pets for the same area and period of study were collected mainly from SIRVERA.

Rabies cases were categorized and analysed using the following host categories: humans, bats, pets, livestock, and wildlife other than bats. Bats were treated as a separate category to highlight and to better analyse the specific role they play in rabies cycle in South America. Throughout the paper, all the data and analysis presented for “wildlife” always refers to “wildlife other than bats”.

### 2.3. Source of Infection for Human Cases

Antigenic typing of rabies viruses using monoclonal antibodies allows us to distinguish trends of disease dissemination and infer the source of infection, as antigenic variants of rabies virus are associated with different rabies cycles and species of terrestrial carnivores and bats in the Americas [24]. However, a spillover of rabies virus into other mammalian species may occur, especially during epizootics [25].

The results of laboratory assays to determine the antigenic variant in human cases are reported through SIRVERA. Even when the source of human infection cannot be determined through laboratory tests, an epidemiological survey is always performed to identify the source of infection for the reported human case. The reporting of human and animal cases of rabies is mandatory in South American countries in compliance with international standards and regulations, WHO’s International Health Regulations 2005 [5], and OIE’s Organic Statutes and standards [18].

Table 1 shows the main rabies antigenic variants identified in the study area (source SIRVERA). Antigenic variants are linked with different animals involved in rabies maintenance and transmission.

The frequency for human cases and the antigenic variants associated, was calculated for each country during the period 2009 to 2018. The percent of source of infection for each country was then calculated.

### 2.4. Dynamics of the Regional Number of Rabies Cases in South American Countries in Time

The evolution of the yearly number of cases in humans, livestock, pets, and wildlife in Latin America from 2009 to 2018 was analysed. The period has been chosen in order to assess the recent evolution of rabies in human, domestic animals (pets and livestock) and wildlife that may be related to the latest recent epidemiological changes in the Region. The Spearman’s rank test was used to evaluate the correlation between years and number of cases. Hochberg’s correction test was performed to counteract the problem of multiple comparisons. All statistical analyses were performed using R version 3.1.3 [26].

### 2.5. Rabies Case Report Rate at Administrative Divisions Level

The compiled number of cases in humans, bats, pets, livestock and wildlife from 2009 to 2018 was calculated and mapped by the first level administrative division. Considering the different epidemiological roles played by bats, they were analysed separately from the other wildlife species. The Chi square test was applied to evaluate statistical association between the administrative divisions and the number of rabies cases. Spatial analyses were performed using QGIS version 2.16 [27]. A dot density map was used to compare distribution of cases in all the categories. Dot map cartograms create a point pattern of cases while reshaping spatial units, such that spatial area becomes proportional to sample size. Dot map cartograms, as demonstrated by Soetens et al. (2017) [28], are a valuable method for detection and visualization of infectious disease outbreaks, which facilitates informed and appropriate actions by public health and animal health professionals.

### 2.6. Quantitative Analysis of Risk Factors Associated with Human Cases Using Poisson Generalized Linear Mixed Model (GLMMs)

Three Poisson generalized linear mixed models (GLMMs) were built to evaluate the risk factors associated with the number of rabies cases in humans. Multilevel models are routinely used in veterinary epidemiology to properly describe the variance associated with a group level [29,30].

In this analysis, the group level (random effect) considered is the first administrative divisions of the countries. The response variables were the following: total number of human cases of rabies per each administrative division during the study period, and number of human cases of rabies per each administrative division due to terrestrial variants during the study period, and number of human cases of rabies per each administrative division due to aerial variants during the study period. Table 2 shows the predictors included in the analysis.

First, the Variance inflation factors (VIFs) (threshold = 5) were considered to detect possible correlation among the predictors. Secondly, the best model was selected using a stepwise approach to minimized Akaike’s information criterion and the likelihood ratio test was performed to evaluate whether the response differs significantly by the random effect variable. The goodness of fit of the models was assessed computing the R^2^ (marginal and conditional R^2^). Finally, overdispersion was checked by visual inspection of the residuals and simulating new data from the fitted model, and then comparing the observed data to those simulated. All the analysis were done using R software version 3.5 (R Core Team, 2018) [26].

## 3. Results

### 3.1. Dynamics of the Regional Number of Rabies Cases in Latin America Countries in Time

A total of 192 human cases were reported during the period of study. The cumulated number of human cases ranged between one and 90 per country, with the highest number reported by Peru. The number of cases recorded in humans varies from six to a maximum of 38 per year, with an average of 19 cases per year.

Rabies cases reported in pets (dogs and cats) were 2740, and 73.5% of them occurred in Bolivia (2015), 11.4% in Brazil (313), 9% in Peru (249) and 1.2% in Venezuela (95). Very few cases (65 in total) were reported in Argentina, Colombia, and Paraguay. The disease was also reported in wildlife with 2143 cases, 93% of them in bats (n = 1990), while other cases in wildlife were registered in South American and crab-eating foxes (n = 111), squirrel and marmoset monkeys (n = 22), South American camelids (n = 11), three cases in kinkajous, two in coatis, in two deer and in two hares. Further taxonomic details of the affected wildlife species are included in Table 3.

In general, pets and wildlife showed an average number of 274 (min = 100–max = 423) and 214 (min = 49–max = 379) cases per year, respectively.

**Figure 1:**
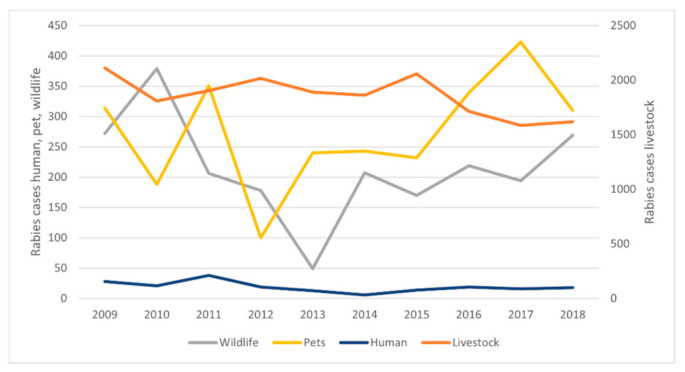
Trends for rabies cases in different animal categories during the period 2009–2018 in South American countries. (Source OIE-WAHIS and SIRVERA).

The disease had its biggest impact on livestock, with 18,568 cases, and an average of 1857 cases per year (min = 1584–max = 2112). The evolution of cases in time in the different categories is represented in Figure 1.

Our analysis showed an overall and significant decrease for the cumulative number of cases (Spearman’s rank-rho = −0.69; adjusted *p*-value < 0.05). The reduction in number of cases was more noticeable for livestock (rho = −0.71; adjusted *p*-value < 0.05), while no significant trend was observed for the other categories.

The categories that registered more fluctuation along the study period are pets and wildlife; in particular, there was a remarkable peak of cases in pets during 2011 followed by a significant decrease in 2012 (almost 70%). Another peak in pet cases was reported between 2016 and 2017, with 339 cases mainly in Bolivia, Peru, and Brazil in 2016 and 423 cases mainly in Bolivia, Peru, Venezuela, and Brazil in 2017. With reference to the cases in wildlife, the lowest values were reported in 2013, followed by a considerable increase up to 2018.

**Figure 2:**
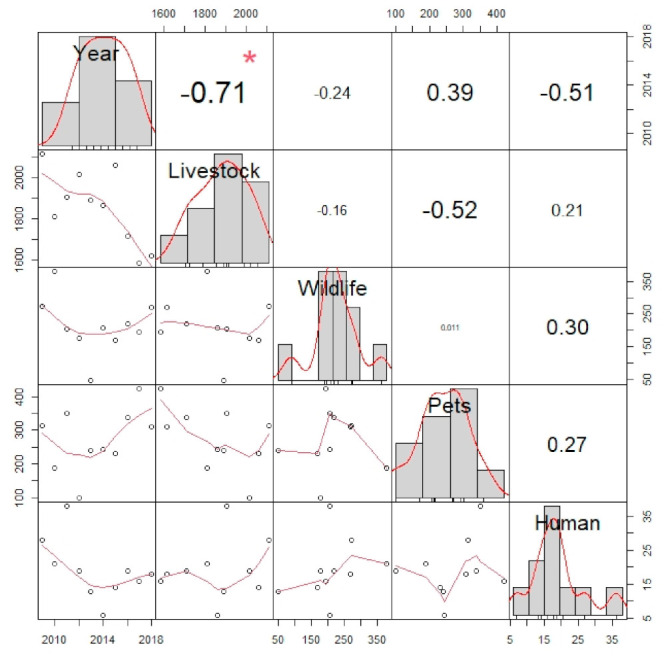
Graph showing the correlation between the number of cases in the different groups of species and over time. The numbers on the right side of the graph represent the correlation coefficient. An asterisk close to the numbers indicates a significant correlation (*p* < 0.05). Data distribution for each category is represented by mean of bar charts. The relation between a pair of variables is represented on the left side of the graph.

Finally, the correlation among the number of cases in the different categories is displayed in Figure 2.

### 3.2. Rabies Case Report Rate at Administrative Divisions Level

From 2009 to 2018, rabies cases were reported in 67.5% (162) of the 239 administrative divisions included in this study. Livestock cases were reported in 59% (141) of the 239 administrative divisions, mainly at latitudes ranging between −35 and 12 degrees. Inside this latitudinal range rabies cases were quite widespread but not homogeneously distributed, with two main clusters in the north-western (mainly Colombia) and south-eastern part of the affected area (Brazil), and a third smaller cluster in Peru. The disease was reported in wildlife in 62 administrative divisions (26% of the administrative divisions included in the study). Most of the cases were detected in bats in 54 administrative divisions (22% of the administrative divisions). Forty-six administrative divisions (19%) reported cases in pets (dogs and cats).

Human cases distribution is more restricted and was reported in 39 (16%) administrative divisions of six countries, with a main cluster in Peru and other cases in Brazil, Bolivia, Ecuador, Colombia, and Venezuela.

**Figure 3:**
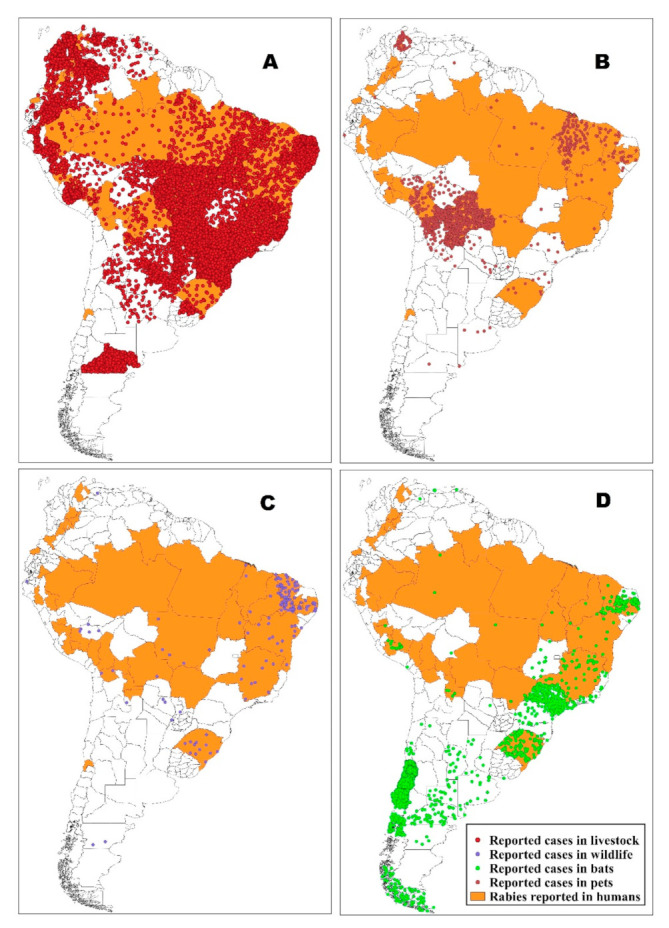
Distribution of the reported rabies cases, represented by dots, for 10 years (2009 to 2018) in livestock (**A**), pets (**B**), wildlife other than bats (**C**), bats (**D**), and comparison with rabies cases reported in humans represented by orange areas. The distribution of the cases is represented using the “density dot” technique.

A strong association was observed between the spatial distribution of affected administrative divisions for human and livestock (Chi square = 20.2; *p* < 0.001; OR = 25.2; IC OR = 3.4–188.8), index of a common risk factor shared by the two categories. Distribution of human, bats, pets, livestock, and wildlife (other than bats) cases is shown in Figure 3.

### 3.3. Source of Infection for Human Cases

In our study, 192 human cases (from seven countries) accounted for the public health burden of the disease as shown in Table 4.

Almost 70% of the human cases were due to contact with wildlife species. The aerial cycle being the most prevalent and the common vampire bats were the main source of infection (134 cases) and accounted for 84 cases in Peru, 11 cases in Ecuador, 9 cases in Colombia and 29 cases for Brazil. As for the sylvatic cycle, there were also 4 human cases attributable to monkey bites in Brazil. In addition, Brazil reported a human case whose origin was probably due to contact with saliva of deer. Regarding the urban (terrestrial) cycle, dogs were the source of infection for 40 human cases in Bolivia, 9 in Brazil, 6 in Peru, 2 in Venezuela and 1 in Chile, whereas cats were the source of infection for 1 human case in Brazil, and 1 in Ecuador.

### 3.4. Quantitative Analysis of Risk Factors Associated with Human Cases Using POISSON Generalized Linear Mixed Models (GLMMs)

None of the four independent variables used in the GLMM showed collinearity problem, so they were all included in the process of model building. The reduced models of each outcome variable are presented in Table 5. The likelihood ratio test for the random effects were statistically significant (*p* value < 0.05) for all the models, implying that the differences among the countries contribute meaningfully to the models. Details on the models’ selection are provided in the Supplementary Material and available online at https://www.mdpi.com/article/10 .3390/tropicalmed6020098/s1.

As shown by Table 4, the mean number of human cases was 1.21 (95%CI 1.15–1.26) times higher for each additional semester in which cases in livestock occurred, and 1.18 (95%CI 1.12–1.24) times higher for each additional semester in which cases in pets occurred. During the study period, there was a statistically significant increase of 76% in the expected number of human cases per each administrative division due to terrestrial variants associated with the six-monthly occurrence in pets. Eventually, the expected number of human cases per each administrative division due to aerial cycle variants increased significantly by 20% for each additional semester in which cases in livestock occurred, and by 16% for each additional semester in which cases in wildlife were detected. In all the models, the magnitude of the variability of the response due to the random effects was substantial.

## 4. Discussion

### 4.1. Rabies as a Public Health Threat

To our knowledge, this is the first study to evaluate the global epidemiological situation of rabies in South America with an integrated approach using the two most important international and regional surveillance systems for human and animal health. The method presented in this work is in line with the recommended One Health approach, providing a meaningful and complete information about the disease, from a human, animal, and environmental perspective. International reporting of countries of the animal epidemiological status through OIE-WAHIS, is quite accurate but can sometimes lack information more easily collected by a regional reporting system. As the National Veterinary Services provide data to the OIE, OIE-WAHIS remains a valuable source of information for livestock and wildlife rabies cases. However, underreporting is expected for human and pets cases. Data from SIRVERA has been previously proven to be particularly useful to assess the epidemiological situation of rabies in South America, particularly for human cases and cases in pets, as these data is usually reported by the Ministry of Health [9]. Nevertheless, the combination of both human and animals’ surveillance systems gives better results, obtaining a more accurate and precise understanding on the real distribution of the disease. It is however important to highlight that, rabies being a neglected disease, with different capacity of countries in detecting and reporting all cases, above all in remote areas, the number of reported cases in animals and wildlife may not always represent the real disease burden for some countries or administrative divisions.

The results presented in this study highlight an improved rabies situation in South America, with an overall progressive reduction in the number of reported cases, especially in humans, but still some critical situation due to the circulation of the virus in wildlife (mainly bats), livestock and pets. Our models indicate that the circulation of the virus in animals continues to represent a public health threat. This is demonstrated by the significant association in the expected number of human cases and the occurrence of rabies in pets and livestock in several areas. This finding highlight that, even if the number of human dog-mediated cases is currently decreasing, the potential public health threat is still high, if rabies is not eliminated in pets, as demonstrated by the recent human cases of dogs-mediated rabies reported in Bolivia in 2021 [9].

Bats were the wildlife species mainly involved in human infection in this study, confirming the findings of previous publications [2,10]. In fact, we found that the increase of human cases due to aerial cycle variants is significantly associated with the occurrence of rabies in wildlife and livestock. These results hint that bat attacks on humans and livestock represent both an animal health and public health concern. Indeed, the occurrence of the disease in livestock may be interpreted as a proxy of the risk represented by the circulation of the virus in common vampire bats. The occurrence dynamic of rabies to humans has been changing in the last years, and in 2004, for the first time in the history of the Regional Rabies Elimination Program coordinated by the Pan American Health Organization (PAHO), the number of human cases of rabies transmitted by wildlife (mainly the common vampire bat) exceeded the number of cases transmitted by dogs. Moreover, in the last few years, improvements in the surveillance for rabies by the Veterinarian Services and Ministries of Health of several South American countries has resulted in a more sensitive system for detecting and investigating all bat-transmitted rabies cases, and therefore, the relative frequency of vampire bat-transmitted human rabies cases is expected to increase [11,31].

Apart from highlighting the status of rabies as a public health threat in South America, this study facilitates the understanding of the geographical distribution of the disease in the region and enables the identification of the areas at greater risk of rabies cases in humans from the different cycles. Analysis of the data from units at the first geopolitical level shows that only 16% (39 of the 239) of the administrative divisions (states/departments/provinces) included in the study reported human cases. This is similar to what was found in previous studies; showing that human cases are limited to shanty towns or peripheral areas of large cities, where street dogs are not vaccinated [32,33]. On the other hand, human cases were mainly localized in the Amazon basin (Peru, Ecuador, Brazil, and Colombia), which is consistent with previous studies [34,35,36]. Two well-defined clusters of human rabies cases were identified in this area. The largest one was located in Peru and Ecuador, and it was linked to the aerial cycle (more than 90% of human cases). The second one was located in Bolivia and was related to the urban cycle (100% of human cases) and involved two of the Departments of Bolivia (Santa Cruz and Cochabamba) with the highest human population density.

It is important to consider that most of human cases were detected in areas deprived of health care facilities. Many of the villages in which bats feed on people are populated by workers from extractive industries such as miners, loggers, or gold-searchers, settlements with precarious housing conditions, allowing bats to enter and feed during the night. Accessibility to post-exposure treatment is not guaranteed in these remote areas. Besides, the residents are not usually aware of the risk of rabies in order to seek for health care after exposure to the common vampire bat’s bite [34]. Despite local efforts to avoid human contagion, the higher exposure risks of some communities, their remote and impoverished nature, finite government resources, and poor understanding of viral persistence mechanisms in vampire bats, are still the main barriers to eliminate human dog-mediated rabies in South America.

According to our study, in administrative divisions where the terrestrial cycle is still present mainly due to dogs-mediated rabies, the risk of human infection was 76% higher than in divisions with no cases in pets, both findings are consistent with previous studies [35]. Furthermore, the risk of a human infection due to the aerial cycle rabies is 20% higher if infections in livestock were detected in the same administrative division in livestock.

### 4.2. Rabies in Livestock, a Burden for Agriculture

The distribution of livestock cases is related to the presence of *Desmodus rotundus* populations since the hematophagous bat is the main reservoir and source of infection for livestock. The geographical distribution of rabies cases in livestock is considered to be linked to the very low vaccine coverage and maintenance in areas with a large amount of susceptible cattle, the most affected livestock species [31,34]. Previous studies shown that paralytic rabies was one of the most reported infectious diseases by South American countries in livestock [36]. Reports from the Pan American Health Organization indicated that despite a reduction by 95% in human and canine cases of rabies in the Americas, since the implementation of the plan in 1983, a more coordinated action is needed to fully eliminate this disease [37,38]. The impact of the disease in South America is greater in cattle than in other domestic species and that is strongly related to the enzootic aerial cycle of the disease transmitted by hematophagous bats. As demonstrated in previous studies, livestock paralytic rabies transmitted by the common vampire bat is largely spread throughout the whole continent and bats have also an important epidemiological role in the transmission of rabies to pets [39,40].

From this study, livestock accounted for most rabies cases registered in animals (84%). Similar studies reinforce the fact that vampire bat-transmitted rabies in South America is currently one of the most serious diseases affecting livestock and humans [40].

### 4.3. Rabies in Wildlife Including Bats and Its Relation with Human Cases

Regarding cases in wildlife, our study shows that 91% (1990) of the cases were reported in bats. In wild animals other than bats, 153 rabies cases were reported, 74% of them belonged to South American foxes and crab-eating foxes (family Canidae, mainly Genus *Lycalopex* and *Cerdocyon*). None of these cases in foxes were a source of infection for humans. Concerning the number of cases, the second wildlife group of importance was the Order of the Primates, with 22 cases of monkeys from the families *Cebidae* and *Callitrichidae* (squirrel and marmoset monkeys). Rabies cases were also reported in members of the *Procyonidae* family, two in a coati (*Nasua nasua*) and three in kinkajous (*Potos flavus*), as this rainforest mustelid mammal is also hunted for pet trade, for fur and bushmeat. The remarkable decrease of rabies cases in wild species reported in 2013 can be compared with the decrease in livestock cases. Since the main source of infection for livestock in South American countries is the hematophagous bat, a decrease on the incidence of cases in bats would involve less cases in livestock, but it is important to take into consideration that as a response to outbreaks, usually surveillance for wildlife species is triggered or enhance during rabies outbreaks in livestock, thus the likelihood of identifying infected bats is higher [36]. The detection of rabies cases in wildlife species other than bats is more occasional, not being in place a systematic monitoring.

Our analysis proved that the risk of human cases of rabies is 16% higher when rabies cases were detected in wildlife (particularly in bats). The risk of rabies transmission from the common vampire bat to humans is lower in urban areas; however, the transformation of sylvatic areas into peri-urban areas may increase the contact rate between humans and the common vampire bat. As peri-urban areas encroach into the sylvatic environment, where people develop productive activities for their subsistence economies, it is likely that difficult socio-economic conditions could prevent potentially infected individuals from seeking health care. In addition, it is known that there are difficulties in accessing these areas to carry out the activities of the control programmes [41,42].

The control activities implemented to prevent the occurrence of human cases of rabies transmitted by the common vampire bats are based on the pre-exposure prophylaxis (PrEP) [43] and the control of the common vampire bat populations. This strategy is also carried out for the control of rabies mediated by vampire bat in livestock [44]. It is usually difficult for the health authorities to provide the PrEP in these peri-urban areas of human encroachment on the sylvatic environment, as most of these areas are only reachable through rivers and located in zones with rugged terrain [45,46]. Furthermore, the control of the common vampire bat populations can only be performed at the feeding sources since the identification of vampire bat roosts’ locations in these areas is almost impossible because of the dense tropical forest, and the countless number of caves, surrounding the communities. Thus, the control actions are executed only once the surveillance health system detects the occurrence of the common vampire bat attacks over the human population in these areas [47,48].

Some bat species can adapt to peri-urban or urban areas. This is particularly the case of insectivorous bats, which are abundant in urban areas due to the density of artificial roosts and insects attracted by city lights. Inter-species transmission of the rabies virus between bats occurs, and this is the most probable mechanism of virus circulation in bat populations [49]. The recurrence of the disease in Uruguay in livestock and dogs [50], as well as the increase in the number of outbreaks during the last 5 years in some regions of Peru, Bolivia, Brazil, and Argentina, has been attributed to ecological changes related to the intensification of livestock production, providing the necessary conditions to maintain a high density of colonies of the common vampire bats with artificial roost sites and abundant feeding source [15,51]. The common vampire bat profits from the gregarious instinct and the natural behaviour of cattle which facilitates their feeding, instead of seeking for other sources of blood in wildlife [52,53,54,55,56]. On the other hand, human rabies transmitted by vampire bats is more common in sylvatic environments and related to the encroachment into the rain forest areas. Livestock paralytic rabies transmitted by vampire bats is largely spread throughout the whole continent. Nevertheless, official livestock vaccination campaigns are usually not included in the Veterinary Services’ control programmes. Generally, vaccination campaigns are implemented after the occurrence of an outbreak, in combination with control of wildlife reservoir [38,57].

There have been several attempts to reduce populations of the common vampire bats that have endangered other bat species. However, reducing paralytic rabies in livestock has not been sustainably achieved [58]. Furthermore, the importance of other bat species for ecological balance and diversity must be taken into consideration, like the insectivorous *Molossidae* family species in the urban and suburban areas for the control of insect populations or the *Glossophaginae* family, for pollinating plants. Spill overs of rabies viruses from bats into dogs in South American countries is well documented. Domestic dogs and cats have been the species most affected by bat-associated rabies (rabies virus of aerial cycle), particularly in countries where dog-mediated rabies has been eliminated and mass vaccination of domestic animals has ceased [34].

### 4.4. Prevention and Control Measures

Our study showed that 30% of cases in humans were related to the terrestrial cycle, mainly dog-mediated rabies. The likelihood of human cases was significantly higher (76%) in areas with cases in dogs. As for the number of cases’ trend in time, cases in pets fluctuated more, 351 rabies cases reported in dogs in Bolivia were responsible for the remarkable peaks in 2011 and 2017. Due to the proximity of pets to humans, an increase in the number of cases in pets led to a considerable increase of human cases of dogs-mediated rabies (terrestrial cycle). In order to fight against dogs-mediated rabies, that has been the main public health concern for several years, under the endorsement of the Pan American Health Organization, the countries of the American Region developed the “Plan of Action for the Elimination of Urban Rabies from the Principal Cities of Latin America”. This plan involved a strategy of dog vaccination program that has decreased the circulation of the variant of canine rabies in domestic dogs since the 1960s [38]. The main objective of controlling rabies in major cities was expanded in the 1990s to cover small villages and neglected areas. Since that time, significant progress has been made in the control of canine rabies although the initial goal to eliminate dogs-mediated human rabies by the year 2005 could not be achieved. Our study shows the persistence of dogs and human cases in the region 10 years later.

Despite intense vaccination campaigns, the vaccination of dogs is still not implemented in certain areas, due to financial constraints among dogs’ owners [34,35]. Consequently, in some areas of the region, canine rabies is still a major concern, as it accounted for 351 cases registered for 2011 in Bolivia, mainly attributed to deficiencies in dog vaccination (low vaccination coverage and poor vaccine quality) and social and environmental conditions. Despite the significant reduction in dogs and human cases, rabies continues to be a threat to public health in South American countries. Some studies indicates that human rabies could be a re-emerging disease in some countries with increasing number of cases [57,58].

Regarding the rabies urban cycle, and even though dogs vaccination is a key component of rabies control and prevention programmes, Peru and Colombia have not reported the execution of official dog vaccination campaigns, while Paraguay has only recently started their implementation, even though some of these countries have also reported rabies in humans. Furthermore, in countries which in recent years have stopped the official vaccination campaigns for cats and dogs such as Argentina and Brazil, the transmission of the rabies virus from bats to pets and subsequently to humans is currently of great concern, making the surveillance of bats a crucial control measure. Although one study affirms that a critical proportion of 39–57% should be enough to prevent dog-to-dog transmission of the rabies virus [59], WHO’s official recommendation for immunization coverage or critical proportion of vaccinated dogs is 70% to prevent human cases of dog-mediated rabies and interrupting rabies transmission at its animal source [43,60].

### 4.5. Limitations of the Study

The main limitations of this study are represented by the challenges in compiling sanitary records that may affect the quality and reliability of the data reported. Because of the nature of this disease, most cases usually take place in rural areas where access to healthcare facilities is limited, in addition, information on rabies cases is collected by different institutions making it difficult to obtain a complete picture from a One Health perspective. Usually, the Ministries of Health collect data for human and pets’ cases while the National Veterinary Services, collect data for livestock and wildlife. Another difficulty for a reliable data collection in South American is the remoteness and poor accessibility. Surveillance programs might not be able to collect appropriate information from these areas [61]. This study is not only one of the few studies that shows the evolution of the disease since 2009 in humans and animals, but it also presents the updated and complete situation of rabies in South America, with data compiled through two of the main international reporting platforms for rabies (OIE-WAHIS and SIRVERA).

## 5. Conclusions

Despite its overall decreasing case report rate, the disease is still a serious problem in South America. The control of rabies requires an integrated approach and coordinated efforts from regional, national, and local governments to avoid rabies-related human deaths and to decrease the economic burden of the disease due to cases in livestock. Although the impact of rabies in livestock, and the fact that there are effective vaccines available, official livestock vaccination campaigns are usually not included in the Veterinary Services’ control programmes. Generally, only emergency vaccination campaigns are implemented after the occurrence of an outbreak, in combination with control of wildlife reservoir. Due to the scarcity of resources, the control of bat-mediated rabies is focused mainly on the control of outbreaks. However, a major heterogeneity in the countries’ actions to prevent rabies is evident and there are several factors preventing the success of the control measures, such as outdated legislation, lack of cooperation between countries, lack of implementation of vaccination campaigns in livestock and the decrease in the control of the population of the common vampire bats. The regional program for rabies in production animals that PANAFTOSA is developing for the Latin American countries aims to tackle these difficulties [62].

In order to prevent human rabies cases transmitted by vampire bats, some countries in South America developed guidelines and training for surveillance and control through a holistic approach including risk characterization of the areas with environmental aspects and possible changes in the productive process such as gold mining and deforestation [15,31]. In addition, surveillance systems for cattle were suggested to alert the health sector about the presence of Lyssavirus in animals [63].

Currently, there is a lack of studies to understand host-virus dynamics for rabies or the ecology of these interactions in South America [64]. Additionally, there is insufficient knowledge of viral persistence mechanisms in bats. Besides, South America’s bat species population are poorly known and other potential host reservoirs carrying rabies virus are not reported in all countries [65]. Future studies on these subjects would be welcomed. In particular, as highlighted by our analysis it would be very important to improve and make routine rabies viruses typing, in order to better understand the disease dynamics. The continuous collection of this information in regional and international databases would be of pivotal importance from an epidemiological perspective.

New strategies for compiling systematic information, networking and education are needed. Intense canine vaccination campaigns are a key component towards the elimination of human dog-mediated rabies in South America. Intersectoral collaboration on rabies control activities in agriculture, health, education, and the treasury at local level (municipalities) would be a valuable strategy. Moreover, adequate training of staff of the veterinary services at local level would be enormously beneficial.

## Figures and Tables

**Table 1:** Antigenic variants of rabies virus identified in South American countries.

Epidemiological Cycle	Antigenic Variant	Characteristics of the Virus	Main Carrier or Host-Reservoir Species
Terrestrial cycle	1	Urban cycle	Dogs-Cats
	2	Sylvatic cycle	Dogs-Foxes
Aerial Cycle	3	Paralytic rabies	hematophagous bats (vampire bats)
	4	Non-hematophagous bats viruses	*Tadarida* spp.
	6		*Lasiurus* spp.
	Others		*Histiotus* spp.
			*Myotis* spp.
			*Eptesicus* spp.

**Table 2:** Overall listing of variables considered to build the GLMMs and their description.

**Occurrence in livestock** Number of semesters with cases in livestock: It is the number of semesters when cases in livestock were reported (20 semesters: from 2009 to 2018). Data source: OIE-WAHIS
**Occurrence in wildlife** Number of semesters with cases in wildlife: It is the number of semesters when cases in wildlife species, excluding in bats were reported (20 semesters: from 2009 to 2018). Data source: OIE-WAHIS
**Occurrence in pets** Number of semesters with cases in pets (cats and dogs) (2009–2018): It is the number of semesters when cases in pets (dogs and cats) were reported (20 semesters: from 2009 to 2018). Data source: OIE-WAHIS and SIRVERA
**Occurrence in bats** Number of semesters with cases in bats (vampire bats + others) (2009–2018): It is the number of semesters when cases in bats were reported (20 semesters: from 2009 to 2018). Data source: OIE-WAHIS.

**Table 3:** Rabies cases reported in wildlife at family, genus or species level.

Family	Genus	Common Name
*Phyllostomidae*	*Desmodus rotundus*	Common vampire bat
	*Artibus lituratus*	Great fruit-eating bat
*Molossidae*	*Tadarida brasiliensis*	Brazilian free-tailed bat
	*unidentified*	
*Vespertilionidae*	*Eptesicus brasiliensis*	Brazilian brown bat
	*incognita/unidentified*	
*Canidae*	*Lycalopex griseus*	South American gray fox
	*Lycalopex vetulus*	Hoary fox
	*Lycalopex* spp.	
	*Cerdocyon thous*	Crab-eating fox
	*Vulpes vulpes*	Red fox
*Procyonidae*	*Potus flavus*	Kikanjou
	*Nasua nasua*	Coati
*Cebidae*	*Saimiri sciureus*	Guianan squirrel monkey
	*unidentified*	
*Callitrichidae*	*unidentified*	
*Camelidae*	*unidentified*	
*Cervidae*	*unidentified*	
*Leporidae*	*Lepus* spp.	

**Table 4:** Number of human cases reported during the period of study, main source of infection in reported cases (aerial or terrestrial) and percentage of administrative divisions reporting rabies cases by country.

Country	Number of Rabies (Cases in Humans)	Predominant Cycle with Regards to the Main Source of Infection (Percentage of Human Cases Due to the Main Reservoir in the Specific Cycle)	Percentage of Administrative Divisions with Human Rabies Cases (Number of Affected Administrative Divisions in Brackets)
Peru	90	Aerial cycle: 93.3% (84) Terrestrial cycle 6.7% (6)	40% (10)
Brazil	38	Aerial cycle: 76% (29) Terrestrial cycle: 24% (9)	52% (14)
Bolivia	40	Terrestrial cycle: 100% (40)	66% (6)
Ecuador	12	Aerial cycle: 91.6% (11) Terrestrial cycle 8.3% (1)	8.7% (2)
Colombia	9	Aerial cycle 100% (9)	15% (5)
Venezuela	2	Terrestrial cycle: 100% (2)	4% (1)
Chile	1	Aerial cycle: 100% (1)	6.6% (1)
Total	192	Aerial cycle: 70% (134) Terrestrial cycle: 30% (58)	16.3% (39)

**Table 5:** Results from the three GLMMs. For each model are presented the variable retained by the model (for both fixed and random effect), the relative risk (along with the 95% confidence interval) and the *p*-value Finally the marginal (variance explained by the fixed effects) and conditional pseudo-R^2^ (variance explained by the whole model) are presented.

**GLMM with Poisson Distribution for the Total Number of Human Cases of Rabies per Each Administrative Division during the Study Period**		
**Fixed Variable**	**Relative Risk (95% CI)**	***p*-Value**
Occurrence in Livestock	1.21 (1.15–1.26)	2.0 × 10^−15^
Occurrence in pets	1.18 (1.12–1.24)	3.8 × 10^−9^
Occurrence in bats	0.93 (0.84–1.02)	0.14
**Random Effect**	**Variance**	**S.D.**
Country	16.20	4.02
**Pseudo-R^2^**	**Marginal**	**Conditional**
	0.09	0.98
**GLMM with Poisson Distribution for the Number of Human Cases of Rabies per Each Administrative Division Due to Terrestrial Variants during the Study Period**		
**Fixed Variable**	**Relative Risk (95% CI)**	***p*-Value**
Occurrence in livestock	1.08 (0.99–1.16)	0.056
Occurrence in pets	1.76 (1.53–2.02)	2.21 × 10^−15^
Occurrence in bats	0.51 (0.24–1.10)	0.087
**Random Effect**	**Variance**	**S.D.**
Country	1.45	1.20
**Pseudo-R^2^**	**Marginal**	**Conditional**
	0.58	0.88
**GLMM with Poisson Distribution for the Number of Human Cases of Rabies per Each Administrative Division Due to Aerial Variants During the Study Period**		
**Fixed Variable**	**Relative Risk (95% CI)**	***p*-Value**
Occurrence in livestock	1.20 (1.15–1.26)	2.74 × 10^−15^
Occurrence in pets	0.87 (0.75–1)	0.06
Occurrence in wildlife other than bats	1.16 (1.02–1.34)	0.02
**Random Effect**	**Variance**	**S.D.**
Country	27.5	5.24
**Pseudo-R^2^**	**Marginal**	**Conditional**
	0.05	0.99

## Data Availability

Data can be found and extracted through OIE-WAHIS (https://wahis.oie.int/#/home) (accessed on 3 June 2021); SIRVERA (https://sirvera.panaftosa.org.br/) (accessed on 3 June 2021).

## Supplementary Materials

The following are available online at https://www.mdpi.com/article/10.3390/tropicalmed6020098/s1.
