# Supplementary material for: Evolution of Rabies in South America and Inter-Species Dynamics (2009–2018)
Source: Trop Med Infect Dis. 2021 Jun 9;6(2):98. doi: 10.3390/tropicalmed6020098 (PMC8293400; doi:10.3390/tropicalmed6020098)
Supplement: Supplementary file 1 [file tropicalmed-06-00098-s001.zip › tropicalmed-1221327-supplementary.pdf]

Model Selection Details

Human Cases of Rabies per each Administrative Unit	
**Model Formula**	**AIC**
Null model	716.2
tot_hum ~ occur_livest+occur_pet+ occur_bat +occur_wild+(1 | country)	612.5
tot_hum ~ occur_pet+ occur_bat +occur_wild+(1 | country)	680.3
tot_hum ~ occur_livest+ occur_bat +occur_wild+(1 | country)	636.8
tot_hum ~ occur_livest+occur_pet+ occur_wild+(1 | country)	612.0
tot_hum ~ occur_livest + occur_pet + occur_bat + (1 | country)	610.5
**Human Cases of Rabies per each Administrative unit due to Terrestrial Variants**	
Null Model	234.5
terr_cycle ~ occur_livest+occur_pet+ occur_bat +occur_wild+(1 | country)	108.6
terr_cycle ~ occur_pet+ occur_bat +occur_wild+(1 | country)	109.9
terr_cycle ~ occur_livest+ occur_bat +occur_wild+(1 | country)	185.5
terr_cycle ~ occur_livest+occur_pet+ occur_wild+(1 | country)	111.3
terr_cycle ~ occur_livest+occur_pet+occur_bat +(1 | country)	106.7
**Human Cases of Rabies per each Administrative Unit due to Eerial Variants During the Study Period**	
Null Model	595.8
aer_cycle ~ occur_livest+occur_pet+ occur_bat +occur_wild+(1 | country)	527.1
aer_cycle ~ occur_pet+ occur_bat +occur_wild+(1 | country)	590.2
aer_cycle ~ occur_livest+ occur_bat +occur_wild+(1 | country)	529.4
aer_cycle~ occur_livest+occur_pet+ occur_wild+(1 | country)	525.2
aer_cycle ~ occur_livest+occur_pet+occur_bat+ (1 | country)	529.5

Variance-Covariance Matrix for all Parameters (Fixed and Random Effect)

Model: Human Cases of Rabies per each Administrative Unit

	(Intercept)	occur_livest	occur_pet	occur_bat	cov_country.(Intercept)
[1,]	2.2597148495	-0.0096970447	-0.0008812338	0.0039729917	-4.291857199
[2,]	-0.0096970447	0.0005541615	0.0000262479	-0.0002906874	0.035036172
[3,]	-0.0008812338	0.0000262479	0.0007701030	0.0001642531	-0.003919408
[4,]	0.0039729917	-0.0002906874	0.0001642531	0.0027367088	-0.024627804
[5,]	-4.2918571993	0.0350361723	-0.0039194076	-0.0246278041	26.583598664

Model: Human Cases of Rabies per each Administrative Unit due to Terrestrial Variants

	(Intercept)	occur_livest	occur_pet	occur_bat	cov_country.(Intercept)
[1,]	0.80326783	-0.0215328034	-0.0259548547	-0.0333524150	-0.698333674
[2,]	-0.02153280	0.0015412907	0.0001484313	-0.0007411757	0.024695080
[3,]	-0.02595485	0.0001484313	0.0050936295	0.0078709213	-0.008399637
[4,]	-0.03335242	-0.0007411757	0.0078709213	0.1505975722	-0.032304208
[5,]	-0.69833367	0.0246950800	-0.0083996370	-0.0323042081	1.648082774

Model: Human Cases of Rabies per each Administrative Unit due to Aerial Variants during the Study Period

	(Intercept)	occur_livest	occur_pet	occur_wild	cov_country.(Intercept)
[1,]	5.840415194	-1.186249e-02	-5.437471e-03	0.003028474	-20.85444368
[2,]	-0.011862490	5.371572e-04	2.100018e-05	0.000029163	0.04850682
[3,]	-0.005437471	2.100018e-05	5.467061e-03	-0.003487211	0.01052727
[4,]	0.003028474	2.916300e-05	-3.487211e-03	0.004969458	-0.01029719
[5,]	-20.854443677	4.850682e-02	1.052727e-02	-0.010297188	120.79225166
